# Significant modification of traditional rapid sequence induction improves safety and effectiveness of pre-hospital trauma anaesthesia

**DOI:** 10.1186/s13054-015-0872-2

**Published:** 2015-04-01

**Authors:** Richard M Lyon, Zane B Perkins, Debamoy Chatterjee, David J Lockey, Malcolm Q Russell, on behalf of Kent, Surrey & Sussex Air Ambulance Trust

**Affiliations:** Kent, Surrey and Sussex Air Ambulance Trust, Wheelbarrow Park Estate, Pattenden Lane, Marden, Kent, TN12 9QJ UK; School of Clinical Sciences University of Bristol & University of Stavanger Norway, North Bristol NHS Trust, Bristol, BS16 1LE UK; Centre for Trauma Sciences, Queen Mary, University of London, London, E1 2AT UK

## Abstract

**Introduction:**

Rapid Sequence Induction of anaesthesia (RSI) is the recommended method to facilitate emergency tracheal intubation in trauma patients. In emergency situations, a simple and standardised RSI protocol may improve the safety and effectiveness of the procedure. A crucial component of developing a standardised protocol is the selection of induction agents. The aim of this study is to compare the safety and effectiveness of a traditional RSI protocol using etomidate and suxamethonium with a modified RSI protocol using fentanyl, ketamine and rocuronium.

**Methods:**

We performed a comparative cohort study of major trauma patients undergoing pre-hospital RSI by a physician-led Helicopter Emergency Medical Service. Group 1 underwent RSI using etomidate and suxamethonium and Group 2 underwent RSI using fentanyl, ketamine and rocuronium. Apart from the induction agents, the RSI protocol was identical in both groups. Outcomes measured included laryngoscopy view, intubation success, haemodynamic response to laryngoscopy and tracheal intubation, and mortality.

**Results:**

Compared to Group 1 (n = 116), Group 2 RSI (n = 145) produced significantly better laryngoscopy views (p = 0.013) and resulted in significantly higher first-pass intubation success (95% versus 100%; p = 0.007). A hypertensive response to laryngoscopy and tracheal intubation was less frequent following Group 2 RSI (79% versus 37%; p < 0.0001). A hypotensive response was uncommon in both groups (1% versus 6%; p = 0.05). Only one patient in each group developed true hypotension (SBP < 90 mmHg) on induction.

**Conclusions:**

In a comparative, cohort study, pre-hospital RSI using fentanyl, ketamine and rocuronium produced superior intubating conditions and a more favourable haemodynamic response to laryngoscopy and tracheal intubation. An RSI protocol using fixed ratios of these agents delivers effective pre-hospital trauma anaesthesia.

## Introduction

Rapid sequence induction of anaesthesia (RSI) is the recommended method to facilitate emergency tracheal intubation in trauma patients [1]. It is a complex intervention with significant risks and the procedure is often tailored to the individual patients’ requirements [2,3]. In the pre-hospital and emergency setting, however, a simple and standardised RSI protocol may improve the safety and effectiveness of the procedure, while also providing training and logistic benefits [4-6]. Currently, there is no accepted standard trauma RSI technique and there is wide variation in practice in the UK [7]. Traditional techniques [8] (comprising pre-oxygenation, administration of a predetermined dose of induction agent and suxamethonium, followed by cricoid pressure) and modifications of these technique are used [9].

The overall aim of RSI is to rapidly provide optimal conditions for tracheal intubation, as this is thought to reduce the risk of aspiration - the leading cause of mortality associated with airway management [1]. In trauma patients, a secondary aim is to avoid harmful pharmacological and physiological derangements that may exacerbate brain injury or haemorrhage. These include episodes of hypoxia, hypotension, acute hypertension and elevated intracranial pressure [10-13]. An ideal trauma technique would therefore rapidly provide optimal intubation conditions, allowing a high rate of first-pass intubation success, while reliably attenuating excessive haemodynamic changes in all patients requiring the procedure. The RSI agents used would have a wide margin of safety and the dosing regimen would be straightforward. Furthermore, ideal pre-hospital RSI agents would not require dilution, reconstitution or refrigeration, and would have minimal side effects.

A number of potential RSI agents are available, each with their own benefits and risk of adverse effects [14]. Suxamethonium is the neuromuscular blocking agent that has traditionally been used for RSI [8]. Its major advantage is a rapid onset of action. Suxamethonium also has a short duration of action, which is regarded as a benefit in situations of unanticipated airway difficulty. Rocuronium is an alternative neuromuscular blocker with a rapid onset of action. It has many of the properties of an ideal pre-hospital RSI agent, however, its long duration of action has been a concern. In practice, wake-up of an injured patient following RSI is rare, even if difficulties are encountered [15]. Furthermore, it is recognised that difficult airway management becomes considerably more complicated in a partially anaesthetised patient as suxamethonium paralysis wears off.

In terms of induction agents, thiopentone and propofol have been shown to cause significant hypotension, particularly in hypovolaemic patients [14,16,17]. Ketamine is a haemodynamically stable induction agent with potent analgesic properties [18]. However, ketamine has historically been contraindicated as an induction agent in patients with suspected head injury due to concerns it may worsen outcome by elevating intracranial pressure. This assumption has not withstood scrutiny and recent evidence suggests that ketamine may have a number of beneficial effects in patients with head injury [18-20]. Etomidate has also been popular for its cardiovascular stability but its use is declining, probably because of well-documented adrenal suppression and infrequent use in elective anaesthesia [21]. Additionally, a study from our group showed that although effective at providing satisfactory intubating conditions, an RSI protocol using etomidate and suxamethonium was ineffective at attenuating the haemodynamic responses to tracheal intubation [22]. The addition of an opiate, to attenuate the haemodynamic response, is an established modification of hospital RSI techniques [23]. This modification is less common in pre-hospital practice due to concerns of precipitating hypotension and adding unnecessary complexity to the procedure.

The major differences between existing trauma RSI protocols are the choice and dose of RSI agents. Few studies have compared the effectiveness of different RSI protocols in the pre-hospital setting. The aim of this study was to compare the safety and efficacy of two standardised pre-hospital RSI protocols: a traditional protocol using etomidate and suxamethonium and a modified protocol using fentanyl, ketamine and rocuronium. We hypothesised that 1) rocuronium would produce equivalent intubation conditions to suxamethonium; 2) the addition of fentanyl would result in a more favourable haemodynamic response to laryngoscopy and tracheal intubation.

## Methods

### Study setting

Kent, Surrey and Sussex Air Ambulance Trust (KSSAAT) operate two dedicated helicopter emergency medical service (HEMS) teams that service a population of approximately 4.5 million and undertake approximately 1,500 missions per year. Each medical team consists of a pre-hospital physician and critical-care paramedic. Physicians have a minimum of 5 years postgraduate experience, including a minimum of 6 months hospital anaesthesia training. Paramedics undergo critical-care paramedic training, including theoretical modules on RSI. Prior to independent pre-hospital practice, medical crew undergo an intense training period including structured medical education, training and operational supervision by pre-hospital care consultants. During this period, training is focused to ensure crews are competent at performing safe pre-hospital RSI. The HEMS team adheres to standard operating procedures (SOPs), which govern all aspects of pre-hospital practice, including pre-hospital anaesthesia, and undertake regular simulation training practice in the application of these procedures.

### Study design

We performed a comparative cohort study over two separate 14-month periods three years apart, comparing two cohorts of major trauma patients undergoing pre-hospital RSI by KSSAAT HEMS. Group 1 (July 2007 to October 2008) underwent pre-hospital RSI using a protocol consisting of etomidate and suxamethonium followed by tracheal intubation. Group 2 (February 2012 to March 2013) underwent pre-hospital RSI using a modified protocol consisting of fentanyl, ketamine and rocuronium followed by tracheal intubation. The study was reviewed by the KSSAAT research and development committee and met National Institute for Health Research Institute criteria for service evaluation. Formal research ethics committee approval was waived and individual patient consent was not required.

### Patient selection

All consecutive trauma patients who underwent pre-hospital RSI during the defined study periods were included. For the purposes of this study RSI was defined as the pre-hospital administration of a muscle relaxant drug (suxamethonium or rocuronium). RSI for medical (non-trauma) indications and cases with no monitor printout record of haemodynamic data were excluded.

### Pre-hospital RSI protocol

The decision to anaesthetise a patient is based on an individual on-scene risk-benefit assessment. Indications include actual or impending airway compromise, ventilatory failure, unconsciousness, anticipated clinical course and humanitarian reasons. Prior to induction, the patient’s position is optimised (ideally on an ambulance trolley with 360° access to the whole patient), all necessary anaesthesia equipment is prepared in a standard kit-dump, non-invasive monitoring is commenced and the patient is pre-oxygenated for at least 3 minutes. Preparation is checked against a challenge-and-response checklist. To meet in-hospital monitoring standards, oxygen saturation, heart rate (HR), electrocardiogram and capnography are continuously monitored using a Lifepak 15 portable monitor (Physio-Control, Redmond, WA, USA) and non-invasive blood pressure (NIBP) is measured every 3 minutes.

RSI drugs are pre-prepared in labelled syringes and induction is achieved by administration of a predetermined dose based on estimated patient weight. Following induction, the trachea is intubated with a bougie and a tracheal tube is *railroaded* into position. Correct placement is confirmed clinically, supported by a qualitative colorimetric CO2 detector (Portex CO2 clip, Smiths Medical, Ashford, UK) and by quantitative waveform capnography.

During the last quarter of 2011, KSSAAT changed the RSI drugs used in the pre-hospital RSI protocol from etomidate and suxamethonium to fentanyl, ketamine and rocuronium. Other than this, the RSI protocol remained identical in both cohorts. In group 1, RSI was achieved with etomidate (0.3 mg/kg intravenously (IV)) followed by suxamethonium (1.5 mg/kg IV). Half the etomidate dose (0.15 mg/kg) was administered in patients with haemodynamic compromise and etomidate was omitted in peri-arrest situations. In group 2, RSI was achieved with fentanyl (3 mcg/kg), ketamine (2 mg/kg) and rocuronium (1 mg/kg). This was known as the 3:2:1 regimen. Drugs were all given in rapid succession in the order fentanyl-ketamine-rocuronium. A reduced dose of fentanyl (1 mcg/kg IV) and ketamine (1 mg/kg IV) was administered in patients with haemodynamic compromise. This was referred to as the 1:1:1 regimen. Again, for severely compromised patients there was the option of administering a muscle relaxant only. There was no standard physiological definition of haemodynamic compromise and the option of full or reduced dosing was left to the discretion of the attending HEMS team. In all the protocols the muscle relaxant dose remained constant.

### Data collection

Data are prospectively collected on all KSSAAT patients. This includes a contemporaneously completed patient report form and electronic database (Aerotech, Horsham, UK), and a printout of three-minute interval monitor recordings. Data on patient demographics, injury characteristics, RSI characteristics (including indications, Cormack and Lehane grade, drug doses and number of attempts) and haemodynamic measures were extracted from these sources. The Trauma Audit and Research Network provided injury severity score (ISS) and outcome data.

### Definitions

For the purposes of this study, RSI was defined as the pre-hospital administration of a muscle relaxant drug (suxamethonium or rocuronium). For group 1, a full-dose RSI was defined as the co-administration of >0.2 mg/kg etomidate. For group 2, a full-dose RSI was defined as the co-administration of >2 mcg/kg fentanyl and ≥1.5 mg/kg ketamine.

The haemodynamic response to laryngoscopy and intubation is the acute change in haemodynamics that occurs within seconds of the stimulus, lasting up to 5 minutes after stimulation has ceased [24,25]. The generally accepted anaesthetic objective is to maintain a stable blood pressure within 10 to 20% of baseline levels [26]. Patients with changes outside this are at increased risk of complications [27,28] and acute elevations in blood pressure (>20%) are typically considered hypertensive emergencies [26]. We defined a hypertensive response as a greater than 20% increase in systolic blood pressure (SBP) or mean arterial pressure (MAP) above baseline and a hypotensive response as a greater than 20% reduction in SBP or MAP below baseline. Absolute hypotension was defined as a reduction in SBP to less than 90 mmHg. Similarly, a tachycardic response was defined as a greater than 20% increase in HR above baseline, and a bradycardic response as a drop in HR to less than 60 bpm. These definitions are consistent with other studies investigating the response [29,30].

Baseline heart rate (HR), systolic blood pressure (SBP) and mean arterial pressure (MAP) measurements were recorded prior to RSI and procedural haemodynamics were the first of these measurements recorded during a 5-minute window following successful tracheal intubation. The timing of successful tracheal intubation was defined by the commencement of capnography. Cases where haemodynamic measurements at these two time points were not recorded were excluded from further analysis of that measurement.

### Outcome measures

The primary outcome was intubation success and the acute haemodynamic response (hypertension, hypotension, tachycardia) to laryngoscopy and tracheal intubation. Secondary outcomes were laryngoscopy view and survival to hospital discharge.

### Statistical analyses

Statistical analyses were performed using Prism 6.0 (Graphpad, La Jolla, USA) software. Normal-quartile plots were used to test for normality. Categorical data are reported as frequency (n) and percent (%) and numerical data as median with IQR. Where appropriate, the chi-square (χ^2^) or Fisher’s exact test were used to compare categorical data and the Mann-Whitney *U*-test or Student’s *t*-test were used to compare numerical data. Paired data were analysed using a paired *t*-test. Statistical significance was set as a two-tailed *P*-value of <0.05.

A multivariable logistic regression model was developed to compare patient, injury, and RSI factors associated with mortality. Factors significantly associated with mortality (*P* <0.1) on univariate analysis were included in the model. Results of the logistic regression model are reported as adjusted odds ratio (OR) with corresponding 95% CI. Statistical significance was set as a two-tailed *P*-value <0.05.

## Results

During the two 14-month study periods, a total of 274 injured patients underwent pre-hospital RSI. Thirteen patients (nine from Group 2 and four from Group 1) were excluded because of missing monitor data, leaving 261 patients available for analysis. There were 116 patients in Group 1 (standard protocol) and 145 patients in Group 2 (modified protocol). Patients in Group 2 were slightly older and more severely injured. Apart from this, the two groups had similar baseline characteristics (Table 1).

**Table 1:** **Baseline characteristics of included patients**

**Characteristic**	**Group 1 (n = 116)**	**Group 2 (n = 145)**	***P*** **-value**
Age, years (range)	39 (2 to 99)	45 (3 to 83)	0.031
Gender, male	86 (74%)	102 (70%)	0.579
Mechanism of injury, blunt	112 (97%)	139 (96%)	1.0
Injury severity:			
Injury severity score	22 (13 to 34)	26 (20 to 38)	0.019
Glasgow Coma score	11 (6 to 14)	9 (5 to 13)	0.061
No head injury	19 (16%)	21 (15%)	0.731
Mild head injury	35 (30%)	17 (12%)	0.003
Moderate head injury	16 (14%)	37 (26%)	0.021
Severe head injury	46 (40%)	70 (48%)	0.171
RSI protocol:			
Full dose	77 (66%)	111 (77%)	0.069
Reduced dose	39 (34%)	34 (23%)	-
RSI indication:			
Unconsciousness	61 (53%)	77 (53%)	0.742
Vent failure	18 (16%)	19 (13%)	-
Anticipated clinical course	16 (14%)	18 (12%)	-
Airway compromise	15 (13%)	20 (14%)	-
Humanitarian	3 (3%)	2 (1%)	-
Facilitate injury management	3 (3%)	9 (6%)	-
Bougie used	114 (98%)	143 (99%)	1.0

Data presented as number (%) or median (interquartile range) unless otherwise specified. Group 1 underwent pre-hospital rapid sequence intubation (RSI) using a protocol consisting of etomidate and suxamethonium. Group 2 underwent pre-hospital RSI using a protocol consisting of fentanyl, ketamine and rocuronium.

### Intubating conditions

**Figure 1:**
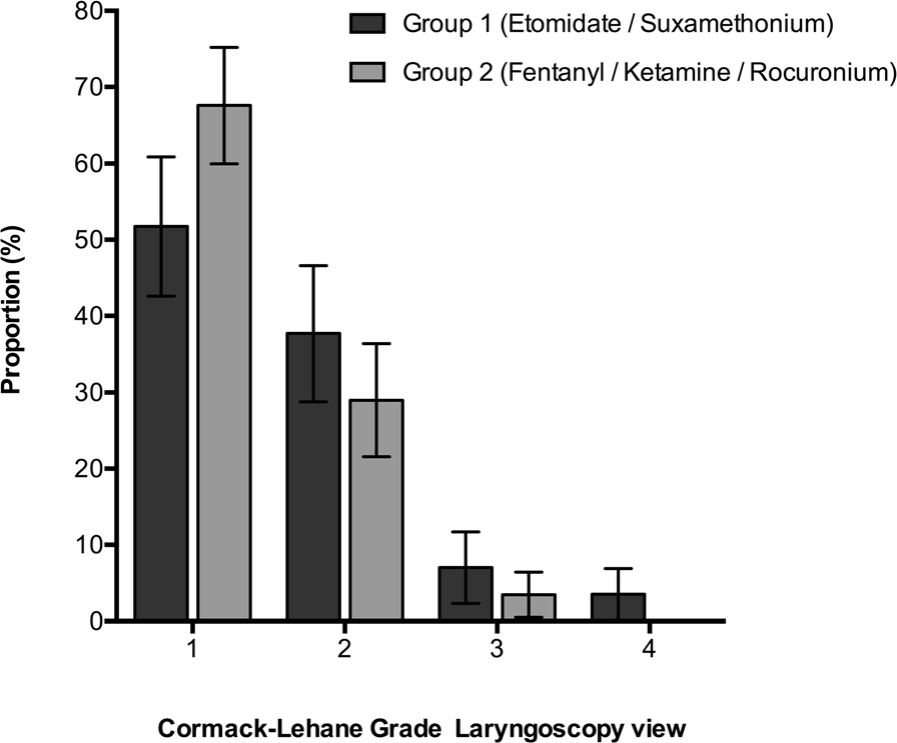
**Cormack-Lehane grade at laryngoscopy by rapid sequence intubation (RSI) group.** Data presented as proportion with 95% CI. There was a significant difference between groups in the proportions (*P* = 0.013, chi-square).

Compared to patients in group 1, laryngoscopy of patients in group 2 resulted in significantly better laryngeal views (*P* = 0.013, chi-square) (Figure 1). In both groups, all tracheal intubations were successful within three attempts. However, first attempt intubation success was significantly higher in group 2 compared to group 1 (95% versus 100%, *P* = 0.007).

### Haemodynamic response to laryngoscopy and tracheal intubation

#### Full-dose RSI

Seventy-seven patients (66%) in group 1 and 111 patients (77%) in group 2 were administered a full-dose RSI protocol. Baseline haemodynamic measures were similar in the two groups (Table 2A). On average, laryngoscopy and tracheal intubation increased HR and blood pressure in all patients (Table 2A).

**Table 2:** **The haemodynamic response to laryngoscopy and tracheal intubation in patients anaesthetised using etomidate/suxamethonium (Group 1) and fentanyl/ketamine/rocuronium (Group 2), stratified by dose administered**

**A) Full-dose RSI protocol**				
	**Baseline**	**Procedural**	**Absolute difference (95% ** **CI)**	***P*** **-value**
**Group 1**				
Heart rate, bpm^a^	89 (72 to 104)	111 (98 to 132)	22 (14 to 38)	<0.0001
Mean arterial pressure, mmHg^b^	98 (90 to 108)	128 (113 to 145)	31 (15 to 48)	<0.0001
Systolic blood pressure, mmHg^b^	129 (114 to 144)	170 (151 to 196)	44 (22 to 61)	<0.0001
**Group 2**				
Heart rate, bpm^c^	87 (74 to 101)	112 (97 to 125)	25 (18 to 30)	<0.0001
Mean arterial pressure, mmHg^d^	102 (90 to 113)	107 (91 to 121)	5 (−1 to 10)	0.148
Systolic blood pressure, mmHg^d^	133 (120 to 149)	140 (120 to 155)	7 (−3 to 11)	0.257
**B) Reduced-dose protocol**				
	**Baseline**	**Procedural**	**Absolute difference (95% ** **CI)**	***P*** **-value**
**Group 1**				
Heart rate, bpm^e^	107 (84 to 121)	122 (111 to 137)	15 (4 to 27)	0.009
Mean arterial pressure, mmHg^f^	79 (54 to 95)	99 (78 to 117)	20 (6 to 36)	0.004
Systolic blood pressure, mmHg^f^	100 (71 to 115)	129 (100 to 147)	29 (13 to 48)	0.001
**Group 2**				
Heart rate, bpm^g^	106 (91 to 131)	123 (105 to 143)	17 (−2 to 28)	0.095
Mean arterial pressure, mmHg^f^	95 (70 to 109)	101 (89 to 126)	6 (−3 to 28)	0.117
Systolic blood pressure, mmHg^f^	117 (104 to 145)	129 (110 to 149)	9 (−7 to 28)	0.256

Data are presented as median (IQR). Analysis based on ^a^74, ^b^ 66, ^c^104 and ^d^95, ^e^36, ^f^24 and ^g^31 patients with complete sets of haemodynamic data. **A)** Full-dose rapid sequence induction (RSI) protocol and **B)** reduced-dose RSI protocol.

The absolute increase in HR was similar in the two groups. The absolute increase in blood pressure, however, was significantly higher in group 1 when compared to group 2 (absolute increase in SBP, 44 mmHg versus 7 mmHg; *P* <0.0001; absolute increase in MAP, 31 mmHg versus 5 mmHg; *P* <0.0001).

**Figure 2:**
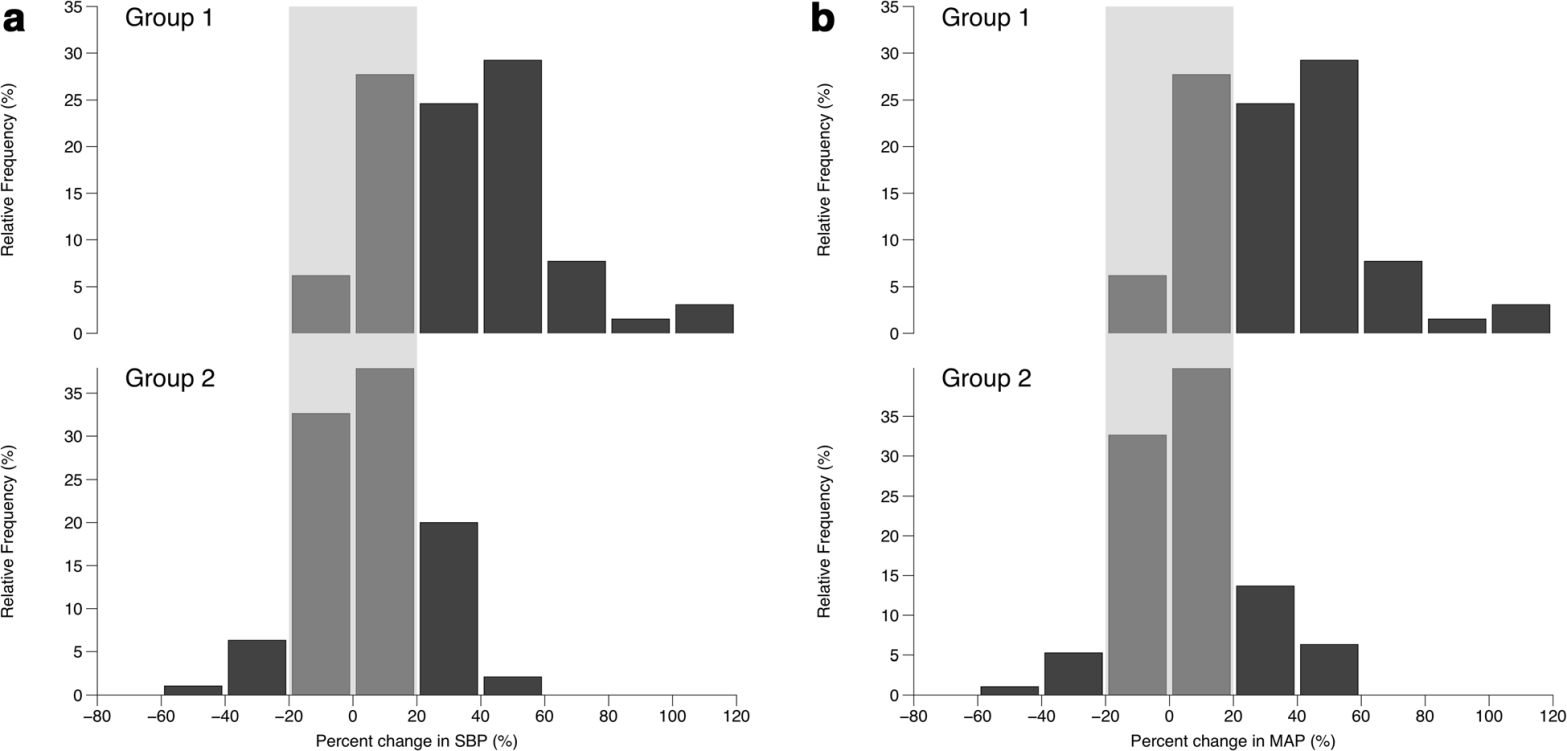
**Relative change in a) systolic blood pressure (SBP) and b) mean arterial pressure (MAP) following a full-dose rapid sequence induction of anaesthesia.** Group 1 were administered etomidate and suxamethonium and Group 2 were administered fentanyl, ketamine and rocuronium. Grey shaded area indicates an acceptable haemodynamic response (within 20% of baseline measurement).

**Figure 3:**
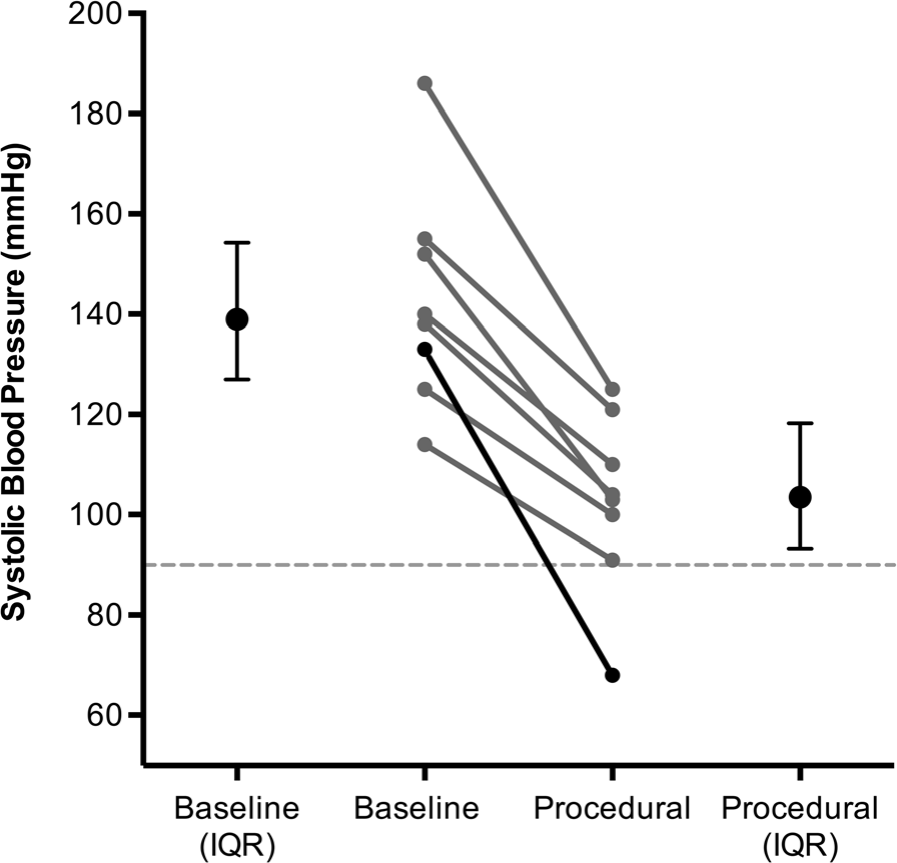
**Baseline and procedural systolic blood pressure (SBP) in the eight Group 2 patients who had a hypotensive response.** The median (IQR) baseline SBP was 140 (127 to 154) mmHg and median (IQR) procedural SBP was 104 (93 to 118) mmHg.

The relative change in blood pressure, following laryngoscopy and tracheal intubation, is shown in Figure 2a (SBP) and Figure 2b (MAP). A hypertensive response was more common following group 1 RSI (80% versus 35%; OR 7.5 (3.6, 15.8); *P* <0.0001) and an acceptable response was most common following group 2 RSI (20% versus 54%; OR 0.22 (0.10, 0.45); *P* <0.0001). Eight patients in group 2 had a hypotensive response (Figure 3) compared to none in group 1(0% versus 7%; OR 0.08 (0.004, 1.4); *P* = 0.022).

#### Reduced-dose RSI

**Figure 4:**
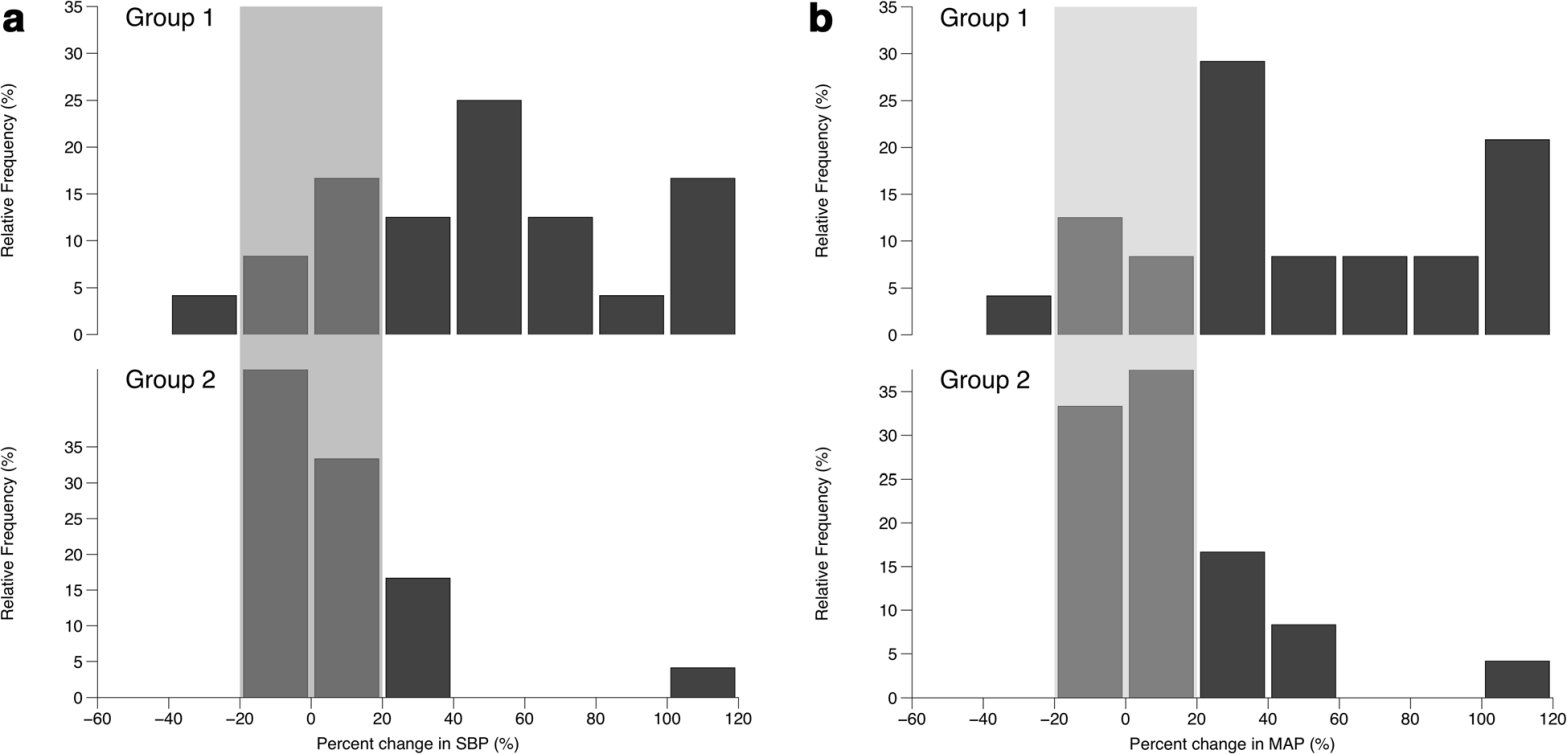
**Relative change in a) systolic blood pressure (SBP) and b) mean arterial pressure (MAP) following a reduced-dose rapid sequence induction of anaesthesia.** Group 1 was administered etomidate and suxamethonium and group 2 was administered fentanyl, ketamine and rocuronium. Grey shaded area indicates an acceptable haemodynamic response (within 20% of baseline measurement).

Thirty-nine patients (34%) in group 1 and 34 patients (23%) in group 2 were administered a reduced-dose RSI protocol. For each group, the haemodynamic response following a reduced-dose RSI was similar to the response observed following a full-dose RSI. On average, laryngoscopy and tracheal intubation increased haemodynamics (Table 2B). The baseline HR and absolute increase in HR following laryngoscopy were similar in the two groups. Baseline blood pressure was significantly lower in group 1 patients and the absolute increase in blood pressure following laryngoscopy was significantly higher in these patients, when compared to group 2 patients (absolute increase in SBP, 29 mmHg versus 9 mmHg; *P* = 0.0008; absolute increase in MAP, 20 mmHg versus 6 mmHg; *P* = 0.013). The relative change in blood pressure, following laryngoscopy and tracheal intubation, is shown in Figure 4a (SBP) and Figure 4b (MAP). The majority of patients in group 1 had a hypertensive response (75% versus 42%; OR 4.2 (1.2, 14.4); *P* = 0.039) whereas patients in group 2 were more likely to have an acceptable haemodynamic response (21% versus 54%; OR 0.22 (0.06 – 0.79); *P* = 0.036). One patient in group 1 had a hypotensive response compared to none in group 2 (*P* = 1.0).

### Acute hypotension

There was a single incident of hypotension in group 1. This patient suffered a blunt, rapid deceleration injury and blood pressure dropped precipitously following tracheal intubation (pre-RSI SBP, 101 mmHg; post-RSI SBP, 68 mmHg). Post-mortem revealed haemorrhage from complete disruption of a major thoracic vascular injury. In group 2, eight patients had a hypotensive response, however, only one patient’s blood pressure dropped below 90 mmHg (Figure 3). This patient suffered blunt chest trauma and developed a tension pneumothorax following tracheal intubation, which was successfully managed with a thoracostomy (pre-RSI SBP, 133 mmHg; post-RSI SBP, 68 mmHg). Half of the patients with a hypotensive response had baseline hypertension (SBP >140 mmHg) that decreased to normotension following induction (Figure 3).

### Outcome

Follow up to hospital discharge was complete for 239 patients (105 in group 1 (91%) and 134 in group 2 (92%)). Overall, 46 patients died from their injuries (19%). In group 1, 20 of 105 patients (19%) died, as compared with 26 of 134 patients (19%) in group 2 (OR 0.98 (0.51, 1.87); *P* = 1.0). Subgroup analysis by head injury severity did not identify any significant differences in outcome.

### Univariate and multivariate analyses

On univariate analysis, the only factors significantly associated with mortality were age, initial Glagow coma score (GCS), injury severity score (ISS), and RSI dose (that is, full or reduced). After adjusting for these variables, only age, initial GCS, and ISS remained independently associated with mortality. The results of the univariate and multivariate analysis are shown in Table 3.

**Table 3:** **Univariate and multivariate analysis of factors associated with mortality in 261 injured patients undergoing pre-hospital rapid sequence induction of anaesthesia by a helicopter emergency medicine service**

**Factor**	**Survived (n = 193)**	**Died (n = 46)**	**Univariate analysis**		**Multivariable analysis**	
			**Crude odds ratio (95%** **CI)**	***P*** **-value**	**Adjusted odds ratio (95%** **CI)**	***P*** **-value**
**Age, years**	39 (23 to 55)	51 (40 to 63)	1.029 (1.012, 1.047)	**0.001**	1.032 (1.006, 1.059)	**0.016**
**Gender**						
Male	140 (80.9)	33 (19.1)	1.041 (0.509, 2.128)	0.913		
Female	53 (80.3)	13 (19.7)				
**Mechanism of injury**						
Blunt	183 (79.9)	46 (20.1)	-	0.216		
Penetrating	10 (100.0)	0				
**Initial physiology**						
Glasgow coma scale	10 (7 to 13)	4 (3 to 10)	0.759 (0.724, 0.874)	**<0.0001**	0.780 (0.680, 0.896)	**<0.0001**
Heart rate	90 (76 to 107)	95 (68 to 125)	1.006 (0.993, 1.018)	0.363		
Systolic blood pressure	127 (112 to 144)	120 (101 to 144)	0.994 (0.981, 1.007)	0.361		
Injury severity score	21 (13 to 34)	34 (27 to 43)	1.077 (1.041, 1.114)	**<0.0001**	1.054 (1.013, 1.098)	**0.009**
**Rapid sequence induction dose**						
Full	148 (86.6)	23 (13.4)	3.289 (1.687, 6.410)	**<0.0001**	1.901 (0.685, 5.272)	0.217
Reduced	45 (66.2)	23 (33.8)				
**Rapid sequence induction protocol**						
ES	85 (81.0)	20 (19.0)	1.023 (0.535, 1.869)	1.000		
FKR	108 (80.6)	26 (19.4)				
Haemodynamic response*						
Normal	79 (88.8)	10 (11.2)	1.541 (0.660, 3.601)	0.317		
Abnormal	82 (83.7)	16 (16.3)				

Data are presented as median (range), number (percent), or odds ratio (95%). Categorical variables are presented with each state on a separate row. The odds ratio represents the odds of survival when the first state (first row) is present compared to when the second state (second row) is present. *Calculated for 187 patients with measurable haemodynamic response. Normal (within 20% of baseline blood pressure), Abnormal (not within 20% of baseline blood pressure). Bold values indicate *P* <0.05. ES, etomidate suxamethonium; FKR, fentanyl ketamine rocuronium.

## Discussion

This study demonstrates the importance of choice of anaesthetic agent in developing a safe and effective pre-hospital trauma RSI protocol. A modified protocol using fentanyl, ketamine and rocuronium produced superior intubation conditions and a more favourable haemodynamic response to laryngoscopy and tracheal intubation when compared to a traditional protocol using etomidate and suxamethonium. Furthermore, in this study ketamine did not appear to have any adverse affects on head injury outcomes, although the study sample size may not have been large enough and may have been prone to type II error. This study suggests that significant departures from traditional RSI protocols can be achieved without major complications and that in our system, RSI using fentanyl, ketamine, rocuronium in a 3:2:1 or 1:1:1 regimen improved the quality of pre-hospital trauma anaesthesia.

In terms of intubation conditions, Perry [31] concluded in a meta-analysis that the use of suxamethonium was associated with superior intubation conditions, but for clinically acceptable intubation conditions there was no difference when compared to rocuronium. In the authors’ subsequent update in 2008, they concluded that rocuronium was inferior to suxamethonium [32]. They did, however, note that the doses of rocuronium in their compared studies varied significantly and called for future studies to look into higher (0.9 to 1.2 mg/kg) doses. In this study higher doses of rocuronium (1 mg/kg) appear to provide superior laryngoscopy views to suxamethonium.

We have previously identified the significant hypertensive response to laryngoscopy and tracheal intubation that occurs following a traditional RSI technique [22]. Furthermore, we observed that following neurotrauma, this response is not attenuated by the depth of coma and massive surges in blood pressure occur unpredictably at all degrees of head injury severity [33]. This is important because even brief episodes of hypertension have been associated with poor outcome following neurotrauma [34]. This study demonstrates that a modified RSI protocol effectively attenuates the haemodynamic response to tracheal intubation.

The safety of ketamine in patients with head injury is gaining acceptance, as there does not appear to be any evidence supporting a harmful effect [20,35]. Instead, emerging evidence suggests that ketamine may be beneficial to patients with head injury and that it may even be the ideal agent for RSI in head injury [18,20,36]. Although our sample size was relatively small and not powered to detect differences in outcome, we did not observe any adverse effect on head injury mortality in patients administered ketamine, despite this group being older and having more severe injuries. We did not examine for emergence phenomena associated with ketamine use.

The combination of fentanyl and ketamine effectively attenuated the hypertensive response to tracheal intubation, however, we observed a group of patients (n = 8) with a greater than 20% drop in blood pressure following this modified RSI. Only one of these patients developed true hypotension (SBP <90 mmHg), which appeared to be caused by a tension pneumothorax rather than being pharmacologically induced. In the remaining patients, the potent analgesic effect of fentanyl and ketamine may be treating pain-induced hypertension, thus, a relative hypotensive response is observed without true hypotension. Etomidate alone has no analgesic properties, and may explain why this effect was not observed following the traditional RSI.

A 3:2:1 or 1:1:1 modified protocol appears safe, although caution should be exercised, particularly in the elderly. Elderly patients may have underlying cardiovascular comorbidities, which poorly tolerate the sympathetic drive of ketamine. This group of patients warrants further investigation. A number of pre-hospital services use an RSI protocol that combines ketamine and a neuromuscular blocker, without an opiate. It is unclear whether this protocol is effective at blunting potentially harmful haemodynamic responses. An analgesic dose of ketamine (average 100 mg), administered prior to traditional RSI, did not appear to blunt the response [22].

This study has several limitations. The study reviewed patients retrospectively rather than conducting a prospective, randomised trial to compare the anaesthetic regimens. The ideal study to accurately compare the two regimens would be a randomised clinical trial, however the ethical, logistical and operational challenges of conducting such a study, together with the sample size needed, were prohibitive to this service at the time. The groups were separated in time. There were some differences in the study groups and this may have influenced the results. However, from a clinical perspective, there were no significant changes in pre-hospital anaesthetic practice other than the RSI agents.

The study included a heterogeneous group of trauma patients. It is possible that differences in pathology may have influenced the study results. Patients in group 2 were older with more severe injuries, potentially causing a bias; however, even in this group, the new RSI regimen appeared safe, although a larger study would be needed to confirm absolute safety. This study only examined the immediate period following RSI and it is possible that subsequent cardiovascular changes may have occurred. Future studies are planned to explore haemodynamic changes during the maintenance phase of anaesthesia. The effect of individual operator variability, particularly when performing intubation, cannot be accounted for, although the use a single SOP may help minimise variation in practice. This study is from a single pre-hospital service and is not powered to detect an effect on patient outcome in terms of survival. Further prospective research is warranted to examine the impact of implementation of this type of modified pre-hospital RSI regimen.

## Conclusion

In this comparative, cohort study, a modified RSI protocol using fentanyl, ketamine and rocuronium provides effective pre-hospital RSI in trauma patients. Using full dose (3:2:1) or reduced dose (1:1:1) regimens appeared to produce superior laryngoscopy views and more favourable physiology during tracheal intubation when compared to a traditional protocol. Further prospective research is warranted to confirm these findings and to examine the outcome of trauma patients undergoing anaesthesia with the modified regimen, including exploring any delayed haemodynamic changes during maintenance of anaesthesia and RSI in the elderly population.

## Key messages

- A simple and standardised RSI protocol may enhance the safety of emergency trauma anaesthesia.
- The choice of anaesthetic agents is an important factor in developing a safe and effective RSI protocol.
- The combined use of fentanyl, ketamine and rocuronium effectively attenuates acute hypertension during pre-hospital intubation, without causing significant hypotension in patients with major trauma.

## Abbreviations

GCS: Glasgow coma scale
HEMS: helicopter emergency medical service
HR: heart rate
ISS: injury severity score
KSSAAT: Kent, Surrey, and Sussex Air Ambulance Trust
MAP: mean arterial pressure
NIBP: non-invasive blood pressure
OR: odds ratio
RSI: rapid sequence induction of anaesthesia
SBP: systolic blood pressure
SOP: standard operating procedure
